# Analysis of Plasmon Loss Peaks of Oxides and Semiconductors with the Energy Loss Function

**DOI:** 10.3390/ma16247610

**Published:** 2023-12-12

**Authors:** Jean-Marc Costantini, Joël Ribis

**Affiliations:** Service de Recherche en Matériaux et Procédés Avancés, Université Paris-Saclay, CEA, 91191 Gif-sur-Yvette, France; joel.ribis@cea.fr

**Keywords:** electron energy loss spectroscopy, energy loss function, dielectric materials, silicon, silicon carbide, cerium dioxide, heavy ion irradiation

## Abstract

This paper highlights the use and applications of the energy loss function (ELF) for materials analysis by using electron energy loss spectroscopy (EELS). The basic Drude–Lindhart theory of the ELF is briefly presented along with reference to reflection electron energy loss (REELS) data for several dielectric materials such as insulating high-k binary oxides and semiconductors. Those data and their use are critically discussed. A comparison is made to the available *ab initio* calculations of the ELF for these materials. Experimental, high-resolution TEM-EELS data on Si, SiC, and CeO_2_ obtained using a high-resolution, double-Cs-corrected transmission electron microscope are confronted to calculated spectra on the basis of the ELF theory. Values of plasmon energies of these three dielectric materials are quantitatively analyzed on the basis of the simple Drude’s free electron theory. The effects of heavy ion irradiation on the TEM-EELS spectra of Si and SiC are addressed. In particular, the downward shifts of plasmon peaks induced by radiation damage and the subsequent amorphization of Si and SiC are discussed. TEM-EELS data of CeO_2_ are also analyzed with respect to the ELF data and with comparison to isostructural ZrO_2_ and PuO_2_ by using the same background and with reference to *ab initio* calculations.

## 1. Introduction

The use of transmission electron microscopy (TEM) and scanning transmission electron microscopy (STEM) with the associated spectroscopies such as electron energy loss spectroscopy (EELS) is nowadays widespread in materials science [1]. It has proven an instrumental means for materials analysis [2]. EELS spectra comprise the low-loss peaks, often assigned to plasmon resonance excitations, and characteristic absorption edges for higher energy losses [1]. In this respect, the home-based ELNES and EXELFS techniques can be considered as competitive with the synchrotron-based X-ray absorption spectroscopy techniques of XANES and EXAFS. Moreover, the combination with other spectroscopies such as Raman scattering and FTIR spectroscopy is also widely used to study materials on different grounds. It is useful to bring about knowledge of the principles of these experimental techniques in order to have theoretical guidelines and fully interpret the data.

For this purpose, we have endeavored hereafter to summarize the method of calculation of the energy loss function (ELF) with the standard Drude–Lindhardt theory and give a critical survey of available data on several dielectric materials used for this calculation. The corresponding *ab initio* ELF calculations are also reviewed to check the relevance of such an analysis. The selected oxides (SiO_2_, Al_2_O_3_, ZrO_2_, TiO_2_, and HfO_2_) are mainly high-k dielectrics for microelectronics devices. Moreover, cerium dioxide (CeO_2_) is envisioned as a solid-oxide fuel cell (SOFC) or solid-oxide electrode cell (SOEC) material [3]. Among semiconductors, silicon and silicon carbide (SiC) play major roles in various technologies, in particular in electronic devices exposed to cosmic ray and solar wind irradiation for space applications [4]. Furthermore, CeO_2_ and SiC are also considered as important materials for nuclear applications: SiC/SiC composites are envisioned as future accident-tolerant fuel cladding [5] and CeO_2_ as a non-radioactive surrogate of actinide dioxides such as PuO_2_ [6] sharing the same cubic fluorite-like structure.

Such a function can also be used for studies of electron-matter or ion-matter inelastic interactions [7]. As such, it is a useful tool for materials scientists in many respects. In the present paper, we highlight the use of the ELF to analyze low-loss peaks as applied to oxides and semiconductors, leaving aside the analysis of absorption edges. Experimental TEM-EELS spectra of virgin and ion-irradiated Si and SiC are discussed using this background. The effect of amorphization on loss peak shift is plainly evidenced for silicon carbide, whereas a much smaller effect is recorded for silicon. The TEM-EELS data of CeO_2_ are also analyzed by using the same background and compared to the ELF data of ZrO_2_, CeO_2_, and PuO_2_ sharing the same cubic fluorite-like crystal structure. A short review of ELF data and *ab initio* DFT calculations for major high-k binary oxides is also included.

## 2. Experimental Procedures

We have used thin epitaxial films of (100) 3C-SiC in thickness of 1.1 µm on a (100) Si wafer in thickness of 500 µm as provided by the NOVASIC Company (Le Bourget-du-Lac, France). Irradiations of SiC films were carried out at room temperature using 2.3 MeV ^28^Si^+^ ions in the JANNUS accelerator (CEA-Saclay, Paris, France) for a flux of 6 × 10^11^ cm^−2^ s^−1^ and in the range of fluences between 1 × 10^14^ and 1 × 10^16^ cm^−2^. The main irradiation features such as ion stopping power and mean projected range (R_p_) with range straggling were computed using the SRIM-2013 code [8]. The R_p_ value (1.24 ± 0.12 µm) in SiC was slightly larger than the film thickness. As a result, ions were implanted in the Si substrate about 400 nm below the SiC/Si interface [9].

Transmission EELS data of virgin and irradiated SiC/Si samples were collected on FIB lamellae with a double-Cs-corrected 200 kV JEOL STEM (neoARM). The energy resolution of the electron gun was of 0.7 eV and the spectrometer resolution was of 0.05 eV per channel. Spectra were taken in the irradiated SiC epilayer and Si substrate for the various fluences with a GIF (Gatan Imaging Filter) of the Continuum type for an energy width of 120 eV and slit aperture of 5 mm. The angle of collection was of 15 mrad. Different kinds of samples were studied: (i) The unirradiated SiC/Si samples; (ii) The irradiated Si substrate and SiC epilayer for the three ion fluences of 1 × 10^14^ cm^−2^, 1 × 10^15^ cm^−2^, and 1 × 10^16^ cm^−2^; (iii) The amorphous a-Si and a-SiC phases. The elastic peak position was shifted to zero-loss if needed. The corresponding TEM images were taken for each fluence to check the microstructure of samples [9]. Measurements were also carried out in the same experimental conditions on a pristine CeO_2_ lamella obtained from a polycrystalline sintered sample, as a test on a reference oxide specimen. The full width at half maximum (FWHM) of the zero-loss peak was of about 1 eV for these three materials.

## 3. Results

**Figure 1:**
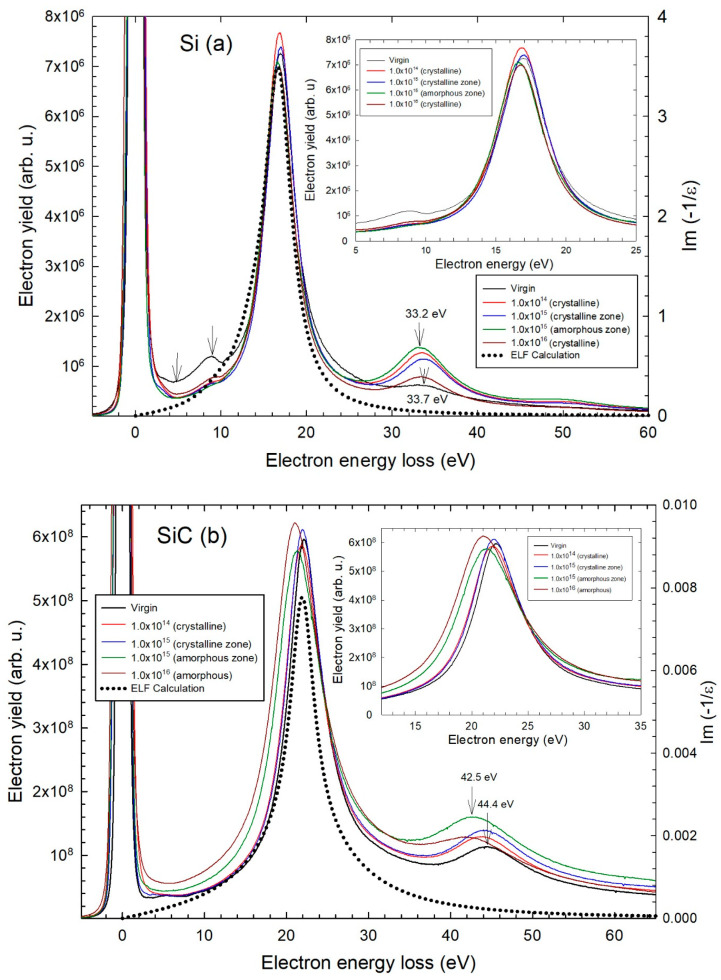
Experimental TEM-EELS data of virgin and irradiated Si (**a**) and SiC (**b**) (solid curves) for the various fluences of 2.3 MeV Si^+^ ions (given in the box in cm^−2^) (left scale) and computed ELF (dotted curves) following Equation (2) by using the parameters of Table 3 (right scale).

All experimental EELS data on SiC/Si samples show the zero-loss elastic peak and the first- and second-order plasmon loss peaks of Si and SiC (Figure 1a,b). The values of the plasmon energy (E_p_) are reported for the various fluences (Table 1). There is a significant downward shift of about 1 eV for the first-order loss peak of SiC near 22 eV, whereas a smaller downward shift of about 0.4 eV is observed for the first-order plasmon loss peak of Si near 17 eV. These data are raw spectra that were not deconvoluted for multiple scattering [1].

HRTEM images were shown for the different fluences in Ref. [9]. It is to be noted that the Si substrate was amorphized in the ion-implanted zone over a thickness of about 400 nm for the fluence of 1 × 10^15^ cm^−2^ and then it was recrystallized after irradiation at 1 × 10^16^ cm^−2^. The SiC epilayer was partially amorphized near the SiC/Si interface over a thickness of about 400 nm for 1 × 10^15^ cm^−2^ and then the epilayer was fully amorphized for 1 × 10^16^ cm^−2^. For the SiC epilayer, there is a significant downward shift of the first-order loss peak of about 1 eV in the amorphous phase for 1 × 10^15^ cm^−2^ and irradiated amorphous phase for 1 × 10^16^ cm^−2^ with respect to the virgin crystal (Table 1). There is also a small downward shift of 0.2 eV for the amorphous SiC phase after irradiation from 1 × 10^15^ cm^−2^ to 1 × 10^16^ cm^−2^. In contrast, for the Si substrate, there is a smaller downward shift of about 0.4 eV of the loss peak of the amorphous phase for 1 × 10^15^ cm^−2^ and about 0.2 eV for the recrystallized substrate for 1 × 10^16^ cm^−2^, with respect to the virgin crystal spectrum (Table 1). The structural states of samples are mentioned for the various fluences in Table 1.

Similar values of the FWHM of ΔE_1/2_~4.3 eV and ΔE_1/2_~5.5 eV are found for the first-order loss peaks of Si and SiC, respectively. No clear variation in FWHM is found versus fluence. The second-order peaks lie at about twice the energy loss value for both materials, yet with larger FWHM values (Figure 1a,b). The same downward shifts are clearly seen even though these peaks arising from multiple scattering are broader than the first-order ones (tagged with arrows in Figure 1a,b, Table 1). There are also smaller peaks near 5 eV and 9 eV (tagged with arrows in Figure 1a) and an asymmetrical broadening of the main loss peak near 17 eV for the virgin Si sample. The elastic zero-loss peak for SiC is also asymmetrically broadened due to a smaller band near 6 eV. The 9 eV peak is seen to decrease with fluence after normalizing the 17 eV peak intensity of Si.

**Figure 2:**
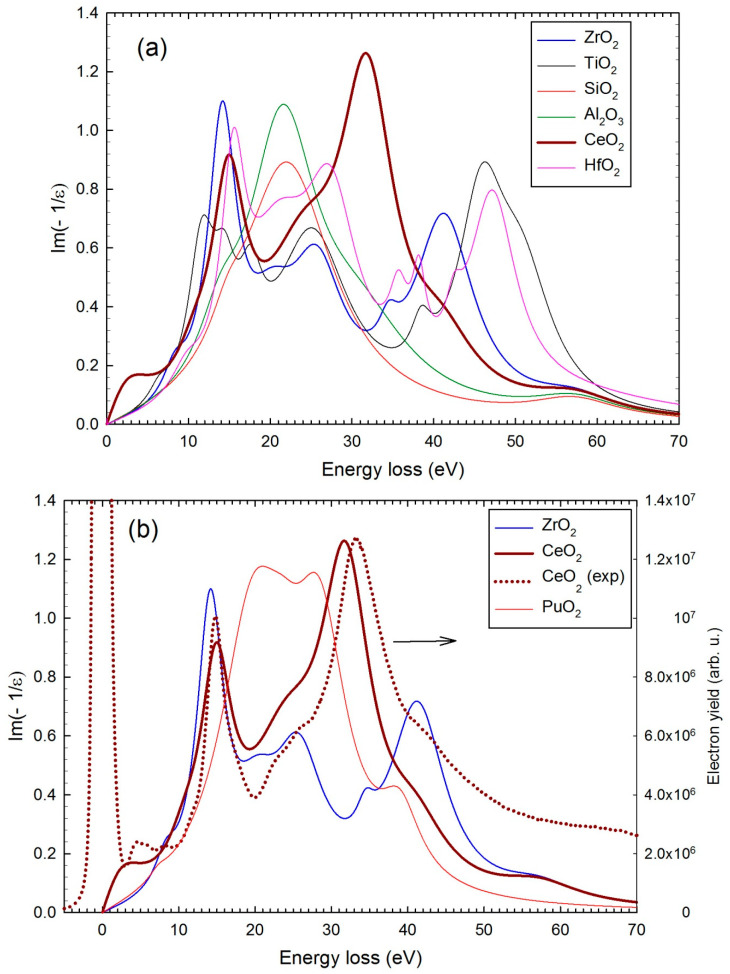
ELF data of six binary oxides (solid curves) computed following Equation (2) by using the parameters of Table 3 (**a**); experimental TEM-EELS data of CeO_2_ (dotted curve; right scale) compared to the computed ELF data of CeO_2_, ZrO_2_, and PuO_2_ (solid curves; left scale) (**b**).

The EELS data of the pristine CeO_2_ sample (Figure 2b) show two strong prominent loss peaks at 14.80 eV with FWHM of ΔE_1/2_~5 eV and 33.40 eV with ΔE_1/2_~12 eV and two secondary broad shoulders near 25 eV and 42 eV. There is also a small peak at 4.75 eV close to the zero-loss peak. The spectrum extends to a broad continuum above 40 eV which is likely due to multiple scattering. TEM images and electron diffraction patterns show a very good crystalline quality with the cubic fluorite structure of CeO_2_.

## 4. Discussion

### 4.1. Background of the Energy Loss Function (ELF)

Surface and bulk plasmon excitations are commonly observed for metallic, insulating, and semiconducting materials, corresponding to the zero values of the real part of the complex dielectric constant $Re\;\left(\epsilon (q,\;\omega \right))$ [10,11]. For insulators and semiconductors, it is known that the plasmon energy (E_p_) is shifted with respect to the free-electron value for metals by an oscillator term corresponding to the band-gap energy (E_G_) [10,12]:

(1) $$E_{p}=\hbar \omega_{p}={\left(E_{G}^{2}+\frac{{\mathrm{ne}}^{2}}{\varepsilon_{0}\varepsilon_{r}{\;m}^{*}}\right)}^{\frac{1}{2}}$$

where *n* is the electron density, ε_0_ and ε_r_ are the dielectric constant of free space and relative dielectric constant of the material, respectively, and e and m* are the electron charge and effective mass, respectively, in the free-electron term $E_{f}=\hbar \omega_{f}=\frac{{\mathrm{ne}}^{2}}{\varepsilon_{0}\varepsilon_{r}{\;m}^{*}}$. Another approach considers an excitonic correction (E_X_) to E_f_ instead of the band-gap energy (E_G_) shift [13].

The loss peaks arising from plasmon resonance appear as maxima of the ELF: Im −1ϵ(q, ω). The Drude–Lindhardt function is commonly used for the calculation of the ELF with a sum of *n* harmonic oscillators:

(2) Im −1ϵ(q, ω)=∑1nfj ħγj ħωħωj,q2−ħω22+ħω ħγj 2 Hħω − EG 

where ħω is the energy loss, f_j_ is the oscillator strength, ħγ_j_ is the width or damping coefficient, ħω_j,q_ is the energy of the jth oscillator, E_G_ is the band gap energy, and *H* is a *Heavyside* step function [14]. The following dependence on momentum transfer (q) is generally applied.

(3) $$\hbar \omega_{j,\;q}=\hbar \omega_{j}+{\;\alpha }_{j}\frac{\hbar^{2}q^{2}}{2m}$$

where α_j_ is the dispersion coefficient depending on the electron effective mass: α_j_ = 1 for free electrons in metals and α_j_ = 0 for wide band gap insulators with flat electron energy bands corresponding to large electron effective masses.

### 4.2. Application to Semi-Conductors

The ELF for silicon deduced from REELS data recorded with low-energy electrons (10 keV) [14] (Table 3) is consistent with the first-order plasmon peaks at about 17 eV in the present experimental TEM-EELS data (Figure 1a). As a first approximation, we have used Equation (2) for calculating the ELF by removing the step function *H*. The latter *Heavyside* step function in general ensures that no electronic excitation would occur below the band gap: i.e., H (ħω − E_G_) = 0, for ħω < E_g_; H (ħω − E_G_) = 1, for ħω ≥ E_G_. Even though α = 0.5 is larger than for the oxides (Table 2), there is no clear impact of the assumption of α_j_ = 0 on the shape and intensity of the prominent ELF peak at 16.8 eV (j = 3) corresponding to the bulk plasmon excitation [14,15]. This volume plasmon resonance is actually found in *ab initio* DFT calculations of the TD-ELF [16]. 

The surface plasmon ELF peak is down-shifted to 10 eV (j = 1) (Table 3) [14,15] which is seen as a smaller band at about 9 eV in the TEM-EELS data of the virgin sample (Figure 1a). There is a clear relative decay of the surface plasmon peak of Si at about 9 eV with respect to the bulk plasmon peak for the irradiated samples. This is likely linked to the ion-induced modifications of the buried SiC/Si epitaxy interface which is not a free surface of the Si substrate.

**Table 3:** Parameters used for fitting the ELF data: oscillator strength (fj), width or damping coefficient (ħγj), energy (ħωj) of the jth oscillator, and tentative assignments of modes. Plasmon peaks are marked in bold.

Material	j	f_j_	ħγ_j_ (eV^2^)	ħω_j_ (eV)	Mode Assignments
**Si**	**1**	**6**	**5**	**10**	Surface plasmon
[14]	2	30	5	14	
	**3**	**210**	**3.8**	**16.8**	Bulk valence plasmon
**SiC**	**1**	**0.46**	**3.5**	**22.0**	Bulk valence plasmon
[17]	**2**	**0.67**	**16.3**	**23.4**	Bulk valence plasmon
	3	0.02	128	158	
**SiO_2_**	1	7.82	5	15	Interband transition
[18]	**2**	**277.91**	**14**	**23.1**	O 2p valence plasmon
**Al_2_O_3_**	1	17.84	6.5	14.3	
[18]	**2**	**209.9**	**10.2**	**22.1**	O 2p valence plasmon
	3	138.47	16	31.8	
**ZrO_2_**	1	2.97	3.5	8.5	O 2p → Zr 4d transitions
[18]	**2**	**59.5**	**4.3**	**14.3**	O 2p valence plasmon
	3	34.71	7	20.5	
	4	94.2	8	26	Collective excitation
	5	11.59	3	34.5	
	**6**	**247.9**	**9**	**41.5**	Zr 4p plasmon
	7	49.58	13	57.5	
**TiO_2_**	1	1.1	3	6.6	O 2p → Ti 3d transition
[19,20]	**2**	**24**	**4**	**11.8**	O 2p valence plasmon
	3	15	3.5	14.5	
	4	17	3.5	17.7	Interband transition
	**5**	**170**	**11**	**25.7**	Collective excitation
	6	17	3	38.5	
	7	210	7	46	
	**8**	**190**	**9**	**51**	Ti 3p plasmon
**HfO_2_**	1	3.9	4.5	10	O 2p → Hf 3d transition/surface plasmon
[21]	**2**	**38**	**3.5**	**15.6**	O 2p valence plasmon
	3	105.4	10	21.5	
	4	140	8	27.7	Collective excitation
	5	22.4	2.8	35.7	Collective excitation
	6	20.9	2	38.2	Collective excitation
	**7**	**14**	**2.4**	**42.5**	Bulk valence plasmon
	**8**	**225.2**	**7**	**47.3**	Zr 4p plasmon
	9	148.7	35	58	
**CeO_2_**	**1**	**3.57**	**8**	**4.55**	Surface plasmon
[22]	2	5.4	5	11	
	**3**	**52.57**	**5**	**15.1**	O 2p valence plasmon
	4	131.62	12	24.7	
	5	265.46	8	32.1	
	**6**	**76.32**	**10**	**41.3**	Ce 5p or Ce 5d plasmon
	7	49.58	13	57.5	
**PuO_2_**	1	0.61	3	7.5	
[23]	2	192.93	10.5	20.38	
	**3**	**26.5**	**6**	**23.9**	Bulk valence plasmon
	4	206	8.7	28.8	Pu 6p_3/2_ excitation
	**5**	**60.89**	**6.56**	**39.1**	Pu 6p or Pu 6d plasmon
	6	20.9	2	38.2	
	7	14	2.4	42.5	
	8	225.2	7	47.3	
	9	148.7	35	58	

Precise measurements using 100 kV STEM for silicon yielded a value of 16.2 eV for the bulk plasmon loss and 8.2 eV for the non-radiative surface plasmon peak of Si with two broad shoulders at about 5 eV and 12 eV [24]. An older conventional 75 kV TEM study gave the values of 16.6 eV and 8.2 eV for the bulk and surface plasmon losses in Si, respectively [24], in agreement with the value of 16.7 eV for the volume plasmon measured using 50 keV electrons in transmission [25]. The second mode at 14 eV (j = 2) for Si induces a broadening of the main plasmon peak of the virgin sample (Figure 1a). For germanium, a similar surface plasmon peak was found at about 15 eV [26,27].

The dispersion relation of Equation (3) is not fully satisfactory in a wide range of q values [25]. Yang et al. have used a Drude-type function but with a different dispersion relation for plasmon excitation:

(4) $${\left(\hbar \omega_{j,\;q}\right)}^{2}={\left(\hbar \omega_{j}\right)}^{2}+\frac{2E_{F}q^{2}}{3}+\frac{q^{2}}{4}$$

where E_F_ is the Fermi energy of the semiconductor. A dispersion relation was also used for the damping coefficient [26] which is generally considered as a constant for the *n* oscillators.

For SiC, a main plasmon peak is found with two almost degenerate oscillator modes at 22.0 eV (j = 1) and 23.4 eV (j = 2) (Table 3) [12,17], which are consistent with the experimental value of 22.1 eV deduced from the present TEM-EELS data (Figure 1b). The first-order plasmon peak is in very good agreement with the calculated spectrum. The third oscillator (j = 3) at 158 eV has a much lower intensity (Table 3) and lies out of the range of the present measurements. The calculated ELF is in agreement with the experimental TEM-EELS spectra for SiC (Figure 1b) which are clearly upward-shifted from the loss peaks of Si (Figure 1a).

The FWHM values of ΔE_1/2_~4.3 eV and ΔE_1/2_~5.5 eV are found for the main loss peaks of Si and SiC, respectively, in good agreement with ΔE_1/2_~4.3 eV and ΔE_1/2_~4.6 eV, as deduced from the calculated ELF, respectively, which is consistent with ΔE_1/2_ = 3.9 ± 0.3 eV for SiC [12]. A smaller value of ΔE_1/2_~3.3–3.4 eV was found using TEM measurements for Si [24]. These FWHM values are clearly larger than the experimental resolution of ΔE~1 eV deduced from the FWHM of the zero-loss peak. The larger FWHM value for the plasmon loss peak of SiC likely stems from lattice imperfections in the strained epilayer due to the large lattice parameter mismatch by about 20% between film and substrate. A high concentration of stacking faults was actually evidenced by HRTEM in the virgin films [9]. However, no significant variations in the FWHM values with ion irradiation are found.

The observed downward shift in the plasmon loss peak by ~1 eV for amorphous SiC can be analyzed on the basis of Equation (1), where E_p_ is dependent on E_G_ and the free-electron density (*n*). A similar downward shift of 1.3 eV of the plasmon peak at 22.2 eV was found at the nuclear-collision damage peak for 4H-SiC after 100 keV He^+^ ion irradiation [28]. A similar shift from 22.4 eV to 19.8 eV was also recorded after the amorphization induced by 12 keV He ion irradiation at 22 K and a smaller decrease to 21 eV at room temperature [29].

It is known that amorphization of semiconductors in general generates a band-gap shrinkage arising from band tailing due to atomic disorder [30]. Actually, a strong decrease in E_G_ from 3.2 eV to 0.5 eV was measured using UV-visible absorption spectroscopy for the 4H and 6H-SiC polytypes [31]. We surmise that a similar band-gap decrease in the band gap energy of the 3C polytype (E_G_ = 2.36 eV) [32] would induce a decrease in E_p_. The free-electron value term ($\hbar \omega_{f}$) is also expected to decrease since the atomic density of the amorphous phase is generally lower than that of the parent crystal. The relative variation in mass density is of about −11–12% for 6H-SiC after amorphization [33,34], yielding a decrease of the electron density (*n*). The volume swelling of 4H-SiC was actually estimated as a function of depth using the shift in plasmon peak for 4H-SiC after 100 keV He^+^ ion irradiation [28]. A similar density decrease in 3C-SiC can also be assumed. Combining these two factors yields a downward shift in the plasmon peak for a-SiC.

More specifically, the plasmon energy can be calculated from Equation (1) for virgin and amorphous SiC. We have taken values of E_G_ = 2.36 eV, *n* = 4.83 × 10^28^ e^−^ m^−3^ (from the Si–C bond density deduced from the mass density of ρ = 3.21 × 10^6^ kg m^−3^ and *p* = 1 e^−^ per bond), and ε_r_ = 9.72 for crystalline 3C-SiC [32]. Moreover, the calculation must take into account the effective mass m* in Equation (1) which is usually deduced from the transverse (m_t_*) and longitudinal (m_l_*) effective masses. Different equations are often used by considering the effective mass either for the electronic density of states or for electrical conductivity. In the present case, m* may be simply deduced as the mean quadratic value of m_t_* and m_l_* as

(5) $$m^{*}=\sqrt{m_{t}^{*2}+{\;m}_{l}^{*2}}.$$

The values of m_t_* = 0.25 m_e_ and m_l_* = 0.50 m_0_ were used for the calculation of E_p_ using the standard MKSA values of the electron rest mass (m_0_) and free-space permittivity (ε_0_), after conversion into eV. These effective mass values are actually near the literature data of m_t_* = 0.25 m_e_ and m_l_* = 0.68 m_0_ for electrons in 4H-SiC [35] and m_t_* = 0.25 m_e_ and m_l_* = 0.67 m_0_ for electrons in 3C-SiC [36]. The calculated value of E_p_ = 22.13 eV matches the experimental value of 22.1 eV. Assuming a decrease in *n* by 15% (*n* = 4.11 × 10^28^ e^−^ m^−3^ deduced from ρ = 2.73 × 10^6^ kg m^−3^ and *p* = 1 e^−^ per Si–C bond) and the same decrease in band gap to E_G_ = 0.5 eV for a-SiC, as for the hexagonal polytypes [31], one finds E_p_ = 20.43 eV by keeping the same values of effective mass (m*) and relative dielectric constant (ε_r_). This is consistent with the decrease by about 1 eV of the plasmon energy for a-SiC. The simple analysis aiming to deduce the change in density of a-SiC by using only the free-electron term is, thus, not correct [28,37,38]. Furthermore, it is seen that modifications still occur in the irradiated amorphous phase when increasing the ion fluence from 1 × 10^15^ cm^−2^ to 1 × 10^16^ cm^−2^, with a downward shift of 0.2 eV which may not be linked to a density decrease only (Table 1).

The smaller downward shift in the plasmon peak for Si arises from the lower mass density change by −1.8% [39]. In contrast to a-SiC, the band-gap value of a-Si (E_G_ = 1.26 eV) [30,40] was found to be somewhat larger than that of c-Si (E_G_ = 1.12 eV) [41]. By following the same procedure of calculation for Si as above for c-SiC, one finds E_p_ = 16.5 eV for c-Si, with E_G_ = 1.12 eV, *n* = 5.39 × 10^28^ e^−^ m^−3^ (deduced from ρ = 2.321 × 10^6^ kg m^−3^ and *p* = 1 e^−^ per Si–Si bond), ε_r_ = 11.7, m_t_* = 0.15 m_e_, and m_l_* = 0.85 m_0_, which is consistent with the experimental value of 17.1 eV. The literature data give near values of m_t_* = 0.19 m_e_ and m_l_* = 0.98 m_0_ for electrons in Si [42]. By applying the decrease in mass density by 2% for a-Si (deduced from ρ = 2.274 × 10^6^ kg m^−3^ and *p* = 1 e^−^ per Si–Si bond), E_G_ = 1.26 eV, m_t_* = 0.16 m_e_, and m_l_* = 0.86 m_0_, one finds the value of E_p_ = 16.2 eV which is consistent with the experimental value of 16.6 eV and the difference of 0.4 eV in plasmon energy from the crystalline to the amorphous phase. This means that there is a compensation of both effects of band-gap energy and free electron density, thereby yielding a very small downward shift in the loss peak for a-Si. It is to be noted that these results are consistent with one free electron per atom in the bulk plasmon band and not the four electrons of the valence band of the semiconductor which would shift the loss peak to twice as much of a value according to Equation (1).

### 4.3. Application to Binary Oxides

We have also used Equation (2) to calculate the ELF for six dioxides by neglecting the step function H. The q-dependence of Equation (2) is also neglected (α_j_ = 0), since it is very weak for these insulators (Table 2). The values of the 3n parameters (f_j_, γ_j_, ħω_j_, j = 1, 2, …*n*) of the *n* oscillators (*n* = 2 for SiO_2_ up to *n* = 9 for HfO_2_) are taken from the literature on the basis of fits of reflection electron energy loss spectroscopy (REELS) data recorded for low electron energies (<2 keV): ZrO_2_ [18,43], TiO_2_ [18,19,20,44], SiO_2_ [14,15,18], Al_2_O_3_ [18,45,46], CeO_2_, [22], and HfO_2_ [21,45] (Table 3). The band-gap energy (E_G_) was also fitted from these experimental data. There are some deviations in the *n* sets of coefficients between the different authors for these oxides. However, the calculated ELFs (Figure 2a) are consistent with the experimental REELS data even without the band gap cut-off that was not included in Equation (2). Even though approximations are very rough when using the simple Drude–Lindhardt function, these calculations convey the major features of EELS data. More complex approaches may be used, such as the full Penn algorithm without any fitting parameter [7,26]. Such an analysis is out of the scope of this paper.

A smooth ELF is found for SiO_2_ with a prominent broad peak of bulk plasmon loss peak at 23.1 eV (j = 2) [14,18], in agreement with other authors [15], which is quite similar to the main loss peak at 22.1 eV (j = 2) [18,45] or 22.7 eV [46] for α-Al_2_O_3_ (Table 3) (Figure 2a). The shoulder below 18 eV (j = 1) for SiO_2_ corresponds to surface excitations and other inter-band transitions [14,15]. The onset of the loss spectrum at about 9 eV for SiO_2_ [15] and Al_2_O_3_ [47] corresponds to electron–hole (exciton) pair formation. TEM-EELS data of Al_2_O_3_ by French et al. highlighted a loss peak at 26 eV [47]. *Ab initio* DFT calculations for α-Al_2_O_3_ gave a bulk plasmon energy of 21.6 eV [48]. However, the other modes at 14.3 eV (j = 1) and 31.8 eV (j = 3) were not accounted for by these calculations.

For ZrO_2_ and HfO_2_, the TEM-EELS data by Frandon et al. show a first peak near 7 or 8 eV corresponding to the first mode of the ELF data (j = 1) which was assigned to excitations of 2p oxygen valence band electrons to the empty d-states [49]. The most prominent peaks at 12 eV for TiO_2_, 14.8 eV for ZrO_2_, and 15.7 eV for HfO_2_ [49] are in agreement with the ELF data (for j = 2) [18,44,45] and were attributed to a bulk valence plasmon mode for a zero value of $Re\;\left(\varepsilon \right)$ [49]. Similar parameters of the oscillators were found by the different authors for these oxides. The peak near 26 eV for ZrO_2_ and HfO_2_ which corresponds to the mode (j = 4) in the ELF data [18,44,45] was also assigned to a “collective excitation” mode of valence electrons.

*Ab initio* TD-DFT calculations have been carried out on the three crystalline phases of ZrO_2_: monoclinic, tetragonal, and cubic [50]. For the cubic phase, the peak at 14.4 eV was assigned to a bulk valence-band plasmon excitation, i.e., the oxygen 2p shell, for $Re\;\left(\epsilon (q,\;\omega \right))$ = 0, while the peak at 24.8 eV was associated to a “collective excitation” and at 41.5 eV to the Zr 4p plasmon excitation also for $Re\;\left(\epsilon (q,\;\omega \right))$ = 0. These computed loss peaks match the major modes for j = 2, 4, and 6, at 14.3, 26, and 41.5 eV, respectively (Table 3). The loss peak at 8.5 eV (j = 1) can be ascribed to electronic transitions from the valence band [50]. These recent calculations are in agreement with the previous assignments by Frandon et al. [49].

Similar *ab initio* TD-DFT calculations of the EELS data were carried out for monoclinic HfO_2_ [51]. The loss peak at 13.5 eV (near the mode for ħω_2_ = 15.6 eV) was the only one to be assigned to a bulk valence band plasmon of mixed O 2p, Hf 5d, and 6s electrons corresponding to a zero value of $Re\;\left(\epsilon (q,\;\omega \right))$. The loss peaks at 13.5 eV and 16 eV were interpreted as surface plasmons and volume plasmons by other DFT calculations [52]. The other features of the computed ELF near 28 eV and 33–37 eV were associated with “collective excitations” and single excitations of Hf 5d electrons, respectively, but not to true plasmons [51,52]. These computed loss peaks are close to the experimental ELF data of ħω_4_ = 27.7 eV, ħω_5_ = 35.7, and ħω_6_ = 38.2 eV (Table 3). The loss peak at 41 eV (near the weak peak at ħω_7_ = 42.5 eV) was interpreted as the total main plasmon of all electrons in HfO_2_ [51], whereas the most prominent peak near 48 eV (near ħω_7_ = 47.3 eV) was analyzed as an Hf 5p plasmon [52]. There is also an overall agreement with the older analysis by Frandon et al. for the peaks at 15.6 eV (j = 2) and 27.7 eV (j = 4) [49]. Similar DFT calculations were carried out for TiO_2_ including Ti 3p semi-core electrons [53]. The computed loss function shows a bulk valence plasmon peak near 10 eV, a collective excitation peak near 25 eV, and a Ti 3p excitation near 50 eV, corresponding to ħω_2_ = 11.8 eV, ħω_5_ = 25.7 eV, and ħω_8_ = 51 eV, respectively [19,20].

Despite some deviations between experimental data for these materials and conflicting interpretations of the ELF data, as displayed up to 70 eV (Figure 2a), the low-loss spectra are quite similar with a prominent peak near 15–20 eV, generally assigned to a O 2p plasmon, except for the strong peak at 33.4 eV for CeO_2_, which may be related to the localized 4f level inside the 2p–5d band gap [54]. The tentative assignments of oscillator modes of the ELF of these binary oxides are given in Table 3. Not all loss peaks can be unambiguously associated to plasmon resonances.

The overall shape of the ELF data for CeO_2_ is in agreement with our TEM-EELS data (Figure 2b) except for a positive shift in this loss peak by about 1 eV with respect to the second maximum of the ELF data corresponding to ħω_5_ = 32.1 eV (Table 3). The FWHM of loss peaks are in agreement with the computed ELF data (Figure 2b). The TEM-EELS peak at 4.75 eV (Figure 2a,b), which is likely a surface plasmon mode, is in agreement with the first oscillator mode at 4.55 eV (j = 1) (Table 3). The two broad shoulders near 25 eV and 42 eV are also consistent with ħω_4_ = 24.7 eV and ħω_6_ = 41.3 eV (Table 3). The weak mode for ħω_7_ = 55.7 eV is broadened in the loss continuum above 50 eV corresponding to multiple scattering (Figure 2b). However, the fitted E_G_ value of 1.5 eV taken by Pauly et al. [46] for the simulations of REELS data is unrealistic, as compared to the experimental photoelectron spectroscopy data by Wuilloud et al. [55] and the UV-visible absorption data by Oh et al. [56] yielding a value of E_G_ = 5.5 eV for the 2p–5d band gap.

We may compare the EELS data of CeO_2_ to ZrO_2_ for the same cubic fluorite structure with similar loss peaks (Figure 2b) (Table 3). The first peak at 14.8 eV for CeO_2_ can be assigned to a bulk valence-band plasmon excitation, i.e., the oxygen 2p shell, like for ZrO_2_ (j = 2, Table 3) [50]. The plasmon energy may be calculated from Equation (1) by taking E_G_ = 5.5 eV [55,56] and ε_r_ = 23 [57]. The electronic density is of n = n_a_ *p*, where n_a_ is the atomic density and *p* is the valence of a given species. For CeO_2.0_, with 8 O atoms per unit cell and a lattice parameter of a_0_ = 0.511 nm, n_a_(O) = 8/(a_0_)^3^ = 5.049 × 10^28^ m^−3^. Hence, n(O) = 1.01 × 10^29^ e^−^ m^−3^, for *p* = 2 e^−^ per oxygen atom. Assuming an isotropic electronic band structure, the values of m_t_* = m_l_* = 0.8 m_0_ are used to compute E_p_ = 14.72 eV, which is in rather good agreement with the loss peak of CeO_2_ at 14.80 eV. These large m* values are consistent with α = 0.2 used for fitting the REELS spectrum [22], corresponding to a rather flat electron energy band: m* is proportional to the radius of curvature of the band extremum, assimilated to a parabolic form, like for standard semiconductors [58]. Such a large m* value is actually consistent with *ab initio* electronic band calculations finding a large radius of curvature of the valence band maximum at the center of the first Brillouin zone (Γ point) [59]. The latter calculations found a direct band gap value of E_G_ = 6.04 eV at the Γ point in rather good agreement with the experimental data corresponding to the O 2p → Ce 5d optical transitions [55,56]. 

The shoulders near 25 eV and 42 eV in the TEM-EELS data of CeO_2_ (Figure 2b) are consistent with the modes at 24.7 eV (j = 4) and 41.3 eV (j = 6) in the ELF data (Table 3). The latter mode may correspond to a Ce 5p or Ce 5d plasmon excitation, as for the Zr 4p plasmon excitation at 41.5 eV (j = 6) in ZrO_2_ [50]. The value of E_p_ = 41.18 eV is obtained by using *p* = 4 e^−^ per Ce atom for 4 Ce atoms per unit cell, i.e., n_a_(Ce) = 4/(a_0_)^3^ = 2.52 × 10^28^ m^−3^, and m_t_* = m_l_* = 0.10 m_0_ for electrons in the 5p or 5d shell. For wide band-gap oxides (E_G_ ≤ 5 eV) with *n*-type conductivity, m* can be computed using *ab initio* DFT/GGA calculations of the electronic band structure, as deduced from the radius of curvature of the conduction band minimum at the Γ point [58]. 

The intermediate prominent loss peak of CeO_2_ at 33.40 eV is close to the much weaker peak at 34.5 eV (j = 5) in ZrO_2_ [18] which is challenging to assess in this simple approach. The difference of about + 1 eV with respect to the ELF data of CeO_2_ on this peak position is puzzling since all the other EELS data are in good agreement with the ELF data by Pauly et al. [22], as mentioned above.

The comparison with the ELF data of PuO*_2_* (Table 3, Figure 2b) may be interesting since CeO_2_ and PuO_2_ share the same cubic fluorite (CaF_2_) structure and the same value of α = 0.2 used for the ELF data analysis (Table 2) [23]. Moreover, both elements Ce and Pu also share the same +3 and +4 oxidation states, with localized 4f and 5f electrons, respectively. There are two prominent loss peaks of PuO_2_ at 20.4 eV (j = 2) and 39.1 eV (j = 5) (Table 3). Even though the latter peak at 39.1 eV may be also assigned to some Pu 6p or Pu 6d plasmon excitation, which is similar to the broad loss peak of CeO_2_ at 41.3 eV (j = 6), the ELF data of these two dioxides look quite different. The peak at 23.9 eV (j = 3) of PuO_2_ could be assigned to a bulk plasmon, whereas the one at 28.8 eV (j = 4) was assigned to the Pu 6p_3/2_ core level ionization from the comparison to the XPS data and not to a plasmon excitation [23]. However, Pu 6p, Pu 6d, and Pu 5f electrons were found to participate in the bonding in PuO_2_ according to electronic structure relativistic calculations on (Pu–O) clusters [60] in agreement with DFT calculations [61]. DFT calculations also found a plasmon excitation at 14.56 eV [60] or 16 eV in PuO_2_ [62].

However, the loss peak at 33.4 eV in the EELS data of CeO_2_ (Figure 2a,b) cannot be associated with the ionization of either Ce 5p_3/2_ or Ce 3s electrons with binding energies of 17.3 eV and 36.7 eV, respectively, as found in the XPS data of CeO_2_ [63]. Electronic structure relativistic calculations on (Ce–O) clusters show that the Ce 4f and Ce 5p do participate to the bonding in CeO_2_ [60]. The tentative assignments of oscillator modes of the ELF of CeO_2_ and PuO_2_ are given in Table 3.

An explanation of the discrepancy between the present TEM-EELS data and the REELS data of CeO_2_ may be linked to the shift in the 4f level in the 2p–5d band gap arising from different populations of this localized electronic level due to the Hubbard-U effect [64]. Actually, Ce^3+^ ions with the 4f^1^ configuration are induced by charge compensation of intrinsic oxygen vacancies in CeO_2–x_, whereas the 4f level of Ce^4+^ is empty for stoichiometric CeO_2.0_. Ab initio DFT calculations have demonstrated a downward shift of about 1 eV between the filled and empty 4f levels [64]. As such, ceria samples may have different oxygen stoichiometries and Ce^4+^/Ce^3+^ redox conditions depending on the methods of preparation, as found, for instance, in XPS data [63]. Differences in oxygen deficiency can be found between the surface and bulk of specimens when comparing the REELS and TEM-EELS data. This shift in 4f level may induce a shift in a loss peak associated with 4f electrons. Moreover, oxygen-atom displacement can occur via elastic collisions for 200 keV electron irradiation [65]. Such an assumption may be tested via measurements of different varieties of ceria obtained in various experimental conditions. From a general standpoint, the comparison of ELF and TEM-EELS data for oxides and semiconductors would need a more rigorous approach using the QUEELS code [66] in order to sort out the surface and bulk excitations and treat the q-dependence of the modes.

## 5. Conclusions

We have reviewed the basic aspects of the Drude–Lindhart theory and the experimental REELS data used for calculations of the ELF. The parameters used for this calculation are presented and critically discussed for seven oxides, namely SiO_2_, Al_2_O_3_, ZrO_2_, TiO_2_, HfO_2_, PuO_2_, and CeO_2_, and for semiconductors such as Si and SiC. A comparison is attempted with experimental TEM-EELS data of virgin and ion-irradiated SiC/Si samples and a virgin CeO_2_ sample recorded at high resolution using a double-C-corrected electron microscope. There is a good agreement with the REELS data on these three crystalline materials except for one of the loss peaks of CeO_2_ near 32 eV with about +1 eV difference in plasmon energy. This discrepancy is attributed to the shift in the Ce 4f level derived from different populations of this level linked to the oxygen deficiency of CeO_2−x_. A clear plasmon peak shift of about −1 eV is observed for amorphous SiC with respect to the pristine crystal, whereas a smaller shift of −0.4 eV is found for the amorphous Si phase. The experimental EELS data are interpreted on the basis of atomic and electronic structure modifications of these semiconductors with a quantitative analysis based on the simple Drude’s free-electron theory. The latter approach is also applied for the quantitative analysis of plasmon loss peaks of CeO_2_.

## Figures and Tables

**Table 1:** Plasmon energies (E_p_) in eV for the first- and second-order loss peaks Si and SiC as a function of the 2.3 MeV Si^+^ ion fluence.

Fluence (cm^−2^)	Peaks	0	1 × 10^14^	1 × 10^15^	1 × 10^15^	1 × 10^16^
**Si**	1st	17.0	16.9 (crystalline)	17.0 (crystalline zone)	16.6 (amorphous zone)	16.8 (crystalline)
	2nd	33.7	33.7	33.7	33.2	33.5
**SiC**	1st	22.1	21.8 (crystalline)	21.9 (crystalline zone)	21.2 (amorphous zone)	21.0 (amorphous)
	2nd	44.4	43.7	43.7	42.5	/

**Table 2:** Parameters of the studied semiconductors and binary oxides: number of oscillators (*n*), dispersion coefficient (α), and band-gap energy (E_G_).

Material	*n*	α	E_G_ (eV)
Si [14]	3	0.5	2.5
SiC [17]	3	0	2.3 (3C)
SiO_2_ [18]	2	0.02	9.3
Al_2_O_3_ [18]	3	0.05	7.1
ZrO_2_ [18]	7	0.05	4.5
TiO_2_ [19,20]	8	0.05	3.0
HfO_2_ [21]	9	0.1	5.5
CeO_2_ [22]	6	0.2	5.5 (*)
PuO_2_ [23]	5	0.2	2.8

(*) E_G_ = 1.5 eV was taken for the simulations by Pauly et al. [22].

## Data Availability

Data may be obtained under request.
